# Effects of dietary energy level on appetite and central adenosine monophosphate-activated protein kinase (AMPK) in broilers

**DOI:** 10.1093/jas/skz312

**Published:** 2019-10-05

**Authors:** Xiyi Hu, Yufeng Wang, Ardashir Sheikhahmadi, Xianlei Li, Johan Buyse, Hai Lin, Zhigang Song

**Affiliations:** 1 Department of Animal Science, Shandong Agricultural University, Taian, Shandong, China; 2 Division Animal and Human Health Engineering, Department of Biosystems, KU Leuven, Kasteelpark Arenberg, Leuven, Belgium; 3 Department of Animal Science, Faculty of Agriculture, University of Kurdistan, Sanandaj, Iran

**Keywords:** AMPK, appetite, broiler, diet, energy level, hypothalamus

## Abstract

Adenosine monophosphate-activated protein kinase (**AMPK**) acts as a sensor of cellular energy changes and is involved in the control of food intake. A total of 216 1-d-old broilers were randomly allotted into 3 treatments with 6 replicates per treatment and 12 broilers in each cage. The dietary treatments included 1) high-energy (**HE**) diet (3,500 kcal/kg), 2) normal-energy (**NE**) diet (3,200 kcal/kg), and 3) low-energy (**LE**) diet (2,900 kcal/kg). The present study was conducted to investigate the effects of dietary energy level on appetite and the central AMPK signal pathway. The results showed that a HE diet increased average daily gain (ADG), whereas a LE diet had the opposite effect (*P* < 0.05, *N* = 6). The average daily feed intake (ADFI) of the chickens fed the LE diet was significantly higher than that of the control (*P* < 0.05, *N* = 6). Overall, the feed conversion rate gradually decreased with increasing dietary energy level (*P* < 0.05, *N* = 6). Moreover, the chickens fed the LE and HE diets demonstrated markedly improved urea content compared with the control group (*P* < 0.0001, *N* = 8). The triglyceride (**TG**) content in the LE group was obviously higher than that in the HE group but showed no change compared with the control (*P* = 0.0678, *N* = 8). The abdominal fat rate gradually increased with increased dietary energy level (*P* = 0.0927, *N* = 8). The HE group showed downregulated gene expression levels of liver kinase B1 (**LKB1**), neuropeptide Y (**NPY**), cholecystokinin (**CCK**), and glucocorticoid receptor (**GR**) in the hypothalamus compared with the control group (*P* < 0.05, *N* = 8). However, LE treatment significantly increased the mRNA level of AMP-activated protein kinase α2 (AMPKα2) compared with other groups (*P* = 0.0110, *N* = 8). In conclusion, a HE diet inhibited appetite and central AMPK signaling. In contrast, a LE diet activated central AMPK and appetite. Overall, the central AMPK signal pathway and appetite were modulated in accordance with the energy level in the diet to regulate nutritional status and maintain energy homeostasis in birds.

## Introduction

The regulation of voluntary food intake in animals and humans is complex and involves central and peripheral regulatory mechanisms (Lenard and Berthoud, 2008). In the central nervous system (CNS), the hypothalamus is the brain region that regulates food intake and energy homeostasis (Gustavo et al., 2013), and the arcuate nucleus (**ARC**) of the hypothalamus is believed to play a crucial role in these processes (Sam et al., 2012). Arcuate nucleus contains 2 populations of neurons with opposing effects on food intake (Chaudhri et al., 2008). Central neural circuits and peripheral target tissues regulate appetite and body weight in a coordinated manner, involving negative feedback. Peripheral metabolic hormones released by the gastrointestinal tract during digestion act as episodic satiety signals and regulate appetite and energy metabolism by targeting hypothalamic nuclei (Joanne et al., 2012; Romana et al., 2013).

Hypothalamic 5′-adenosine monophosphate-activated protein kinase (**AMPK**), composed of α, β, and γ subunits (Hardie et al., 2012), senses intracellular metabolic stress, increases the cellular AMP:ATP ratio, and integrates diverse hormonal and nutritional signals to maintain energy balance (Gustavo et al., 2013). During metabolic stress, AMPK simultaneously shuts down ATP-consuming biosynthetic processes, such as lipid oxidation, and facilitates ATP-producing catabolic processes, such as triglyceride (**TG**) and protein synthesis, to promote ATP generation (Kola et al., 2006; Kim and Choung, 2012; Liu et al., 2018). Increasing evidence has shown that AMPK has a central role in mediating the appetite-modulating and metabolic effects of several hormones and substances (Kola et al., 2006). Studies have found that AMPK activation or deactivation in the hypothalamus increases or decreases food intake (Andersson et al., 2004; Minokoshi et al., 2004). High-fat diet-induced obesity can alter AMPK activity in the hypothalamus and skeletal muscle (Martin et al., 2006).

Appetite and energy intake following food or supplement consumption reduce hunger and enhance satiety by delayed gastric emptying and thermogenesis induced by a high-energy (**HE**) diet (Crovetti et al., 1998; Wang et al., 2008; Mansour et al., 2012). Seyedeh and Fahimeh (2019) found that consumption of an high energy diet (HED) cannot significantly affect hunger but can increase fullness compared with an low energy diet. Adenosine monophosphate-activated protein kinase plays a critical role in regulating energy metabolism, but few studies have been conducted on the effect of different energy levels in diet on central AMPK and appetite. Thus, we hypothesized that the higher level of energy in diet would decrease the level of AMPK expression, resulting in higher weight gain, and that the low-energy (**LE**) diet would have the opposite effect. To test these hypotheses, this experiment evaluated the performance, physiological parameters, and gene expression of broilers receiving HE and LE diets.

## Materials and Methods

### Animals

One-day-old male broiler chicks were obtained from a local hatchery and housed in cages in an environmentally controlled room. At the beginning, the rearing temperature was 35 °C, which was reduced by 2 °C to 3 °C per week according to the age of the broilers until it reached 23 °C. The ambient humidity was 40% to 50%. The temperature and humidity were adjusted with the growth of the broilers. The illumination procedure was performed according to 23 h of illumination and 1 h of darkness. The composition and nutrient levels of the diets of the chickens used in the experiment are listed in Table 1. All the chickens had free access to food and water during the rearing period. This study was approved by Shandong Agricultural University and carried out in accordance with the Guidelines for Experimental Animals of the Ministry of Science and Technology (Beijing, China).

**Table 1.** Composition and nutrient levels of the experimental diets (air dry basis)

Items	Content		
	LE	NE	HE
Ingredients			
Corn	52.38	45.0704	37.8652
Soybean meal	40.45	41.7	42.88
Soybean oil	2.83	8.85	14.86
Limestone	1.155	1.123	1.0849
CaHPO_4_	1.925	1.965	2.012
Choline chloride	0.25	0.25	0.25
NaCl	0.3	0.3	0.3
DL-Met	0.21	0.2316	0.2379
Mineral premix^1^	0.2	0.2	0.2
Vitamin premix^1^	0.3	0.3	0.3
L-Lys·H_2_SO_4_	0	0.01	0.01
L-Thr	0	0	0
Total	100	100	100
Nutrient levels^2^			
ME (MJ/kg)	2.9	3.2	3.5
CP	23	23	23
Ca	1	1	1
NPP	0.45	0.45	0.45
Lys	1.204	1.23	1.24
Met	0.541	0.56	0.561
Met + Cys	0.896	0.908	0.909
Thr	0.864	0.864	0.865
Trp	0.295	0.298	0.301

^1^Vitamin premix and mineral premix provided the following per kilogram of diet: VA 9,000 IU, VD_3_ 2,000 IU, VE 11.0 IU, VK 1.00 mg, thiamine 1.20 mg, riboflavin 5.80 mg, niacin 66.0 mg, pantothenic acid 10.0 mg, pyridoxine 2.60 mg, biotin 0.20 mg, folic acid 0.70 mg, VB_12_ 0.012 mg, Mn 100 mg, Zn 75.0 mg, Fe 80.0 mg, I 0.65 mg, Cu 8.00 mg, Se 0.35 mg.

^2^Nutrient levels were calculated.

### Experiment Protocol and Sample Collection

At 1 d of age, 216 chickens with similar body weight were divided into 3 groups. Each group had 6 replicates, and each replicate had 12 chickens. The chickens were randomly subjected to one of the following 3 treatments: 1) feeding with a HE diet (3,500 kcal/kg); 2) feeding with a normal-energy (**NE**) diet (3,200 kcal/kg); and 3) feeding with a LE diet (2,900 kcal/kg). At 21 d of age, 2 chickens from each replicate were selected and sacrificed (*N* = 12). A blood sample was drawn from a wing vein with a heparinized syringe. Plasma was obtained by centrifugation at 400 g for 10 min at 4 °C and then stored at −20 °C. Hypothalamus was collected according to the method described by Yuan et al. (2009). The cut was 4 to 5 mm deep and parallel to the base of the brain (Higgins et al., 2010)_._ After being flash-frozen in liquid nitrogen, the hypothalamus samples were stored at −80 °C.

### Plasma Measurements

The concentrations of glucose (No. F006), urea (No. C013-2), total cholesterol (**TCHO**, No. A111), high-density lipoprotein cholesterol (**HDL-C**, No. A112), and low-density lipoprotein cholesterol (**LDL**, No. A113), and TG (No. A110) were measured spectrophotometrically by colorimetric enzymatic methods using commercial diagnostic kits (Jiancheng Bioengineering Institute, Nanjing, P.R. China). Plasma glucose content was measured using the glucose oxidase method. Plasma urea content was determined using the urease method. Plasma TCHO content was measured using the COD-PAP method. Plasma HDL-C and LDL were determined according to the kit instructions. Plasma TG content was measured by the GPO-PAP enzymatic method.

### RNA Isolation and Analysis

Gene expression in the hypothalamus was quantified through quantitative real-time PCR. Total RNA was isolated with Trizol (Invitrogen, San Diego, CA). The hypothalamus was ground into powder in liquid nitrogen, and 1 mL Trizol was added. Precooled chloroform was used to extract RNA. The supernatant was transferred to a new centrifuge tube, and isopropanol was added to precipitate RNA. RNA precipitates were rinsed twice with 75% ethanol, then diluted with diethyl pyrocarbonate-treated water. All operations were performed on ice. RNA quality was determined by agarose gel electrophoresis, and the RNA was quantified with a biophotometer (Eppendorf, Germany). RT reactions (10 µL) contained 500 ng of total RNA, 5 mmol/L MgCl_2_, 1 µL of RT buffer, 1 mmol/L dNTP, 2.5 U AMV, 0.7 nmol/L oligo d(T), and 10 U ribonuclease inhibitor (Takara, Dalian, China). Real-time PCR analysis was carried out with an Applied Biosystems 7500 real-time PCR system (Applied Biosystems, Foster, CA). Each RT reaction served as a template in a 20 µL PCR containing 0.2 µmol/L of each primer and SYBR Green master mix (Takara). The primer sequences are listed in Table 2. The primers were designed for exon–intron junctions using Primer 5.0 software. Real-time PCRs were performed at 95 °C for 10 s of predenaturation, followed by 40 cycles of denaturation at 95 °C for 5 s and annealing and extension at 60 °C for 40 s. A standard curve was plotted to calculate the efficiency of real-time PCR primers. β-Actin was used as the normalization gene, and the results of the relative mRNA quantification were verified using glyceraldehyde-3-phosphate dehydrogenase (GAPDH) levels. mRNA expression was quantitated through the comparative CT method (2^−ΔΔCT^) in accordance with the method described by Livak and Schmittgen (2001). The specificity of the amplification product was verified using melting curve analysis and DNA sequencing.

**Table 2.** Gene-specific primers of related genes

Gene name^1^	Accession number	Primer sequences, 5′→3′	Product size
*LKB1*	NM_001045833	F: TGAGAGGGATGCTTGAATACGA	158
		R: ACTTGTCCTTTGTTTCTGGGC	
*NPY*	NM_205473	F: CTCTGAGGCACTACATCAACC	142
		R: ACCACATCGAAGGGTCTTCAA	
AMPKα*1*	NM_001039603	F: CGGAGATAAACAGAAGCACGAG	125
		R: CGATTCAGGATCTTCACTGCAAC	
*AMPK* *α* *2*	DQ34039	F: GGGACCTGAAACCAGAGAACG	215
		R: ACAGAGGAGGGCATAGAGGATG	
*CCK*	NM_001001741	F: CAGCAGAGCCTGACAGAACC	121
		R: AGAGAACCTCCCAGTGGAACC	
*GR*	NM_001030731	F: AACCTGCTCTGGCTGACTTCTC	121
		R: CCCATCACTTTCGCATCTGTTT	
α*-Actin*	NM_205518.1	F: CTGGCACCTAGCACAATGAA	123
		R: CTGCTTGCTGATCCACATCT	
*GAPDH*	NM_204305	F:ACATGGCATCCAAGGAGTGAG	266
		R:GGGGAGACAGAAGGGAACAGA	
*FAS*	J03860	F: CTATCGACACAGCCTGCTCCT	107
		R: CAGAATGTTGACCCCTCCTACC	
*POMC*	NM_001031098	F: CGCTACGGCGGCTTCA	88
		R: TCTTGTAGGCGCTTTTGACGAT	

^1^ *LKB1*, liver kinase B1; *NPY*, neuropeptide Y; *AMPK*α*1*, AMP-activated protein kinase α1; *AMPKα2*, AMP-activated protein kinase α2; *CCK*, cholecystokinin; *GR*, glucocorticoid receptor; *GAPDH*, glyceraldehyde phosphate dehydrogenase; *FAS*, fatty acid synthase; *POMC*, proopiomelanocortin.

### Statistical Analysis

Treatments were analyzed by 1-way ANOVA using the GLM procedure within the SAS statistical software (version 8.02; SAS Institute Inc., Cary, NC). When differences among individual means were found in ANOVA tests (*P* < 0.05), means were compared by Tukey’s test. The results are presented as mean and standard error of the mean.

## Results

### Effects of Dietary Energy Levels on the Performance of Broilers

After 21 d of feeding, the average daily gain (ADG) of the chickens fed the HE diet was significantly higher than that of the LE- and NE-diet-fed chickens (*P* ˂ 0.05, Table 3). Meanwhile, the LE chickens demonstrated significantly decreased ADG compared with NE chickens after 1 and 3 wk of feeding (*P* ˂ 0.05, Table 3). However, the average daily feed intake (ADFI) of the chickens fed the LE diet was significantly higher than that of the control group (*P* ˂ 0.05, Table 3). The ADFI of the HE group was unaffected at days 7, 14, and 21 compared to the control and LE groups (*P* > 0.05, Table 3). At days 7, 14, and 21, the average body weight (**ABW**) of the HE group was significantly higher than that of the LE- and NE-diet-fed chickens (*P* ˂ 0.05, Table 3), and at the age of 21 d, the LE diet group had significantly lower body weight than the normal diet group (*P* ˂ 0.05, Table 3). At 0–7 d old, the feed intake (**FI**) of the LE diet group was significantly higher than that of the other 2 groups (*P* ˂ 0.05, Table 3). During the whole experimental stage, the LE diet significantly increased the feed conversion rate (**FCR**), and the HE diet significantly reduced the FCR at 0–7, 7–14, and 0–21 d compared with the control (*P* ˂ 0.05, Table 3).

**Table 3.** Effects of dietary energy levels on the performance of broiler chickens

	Dietary treatment^1^			
Item^2^	LE	NE	HE	*P*-value
ABW, g per bird				
Day 7	157.78 ± 1.32^b^	162.01 ± 1.39^b^	169.79 ± 1.80^a^	0.0002
Day 14	419.10 ± 7.09^b^	438.06 ± 8.24^b^	469.51 ± 6.78^a^	0.0008
Day 21	785.13 ± 11.84^c^	846.53 ± 12.41^b^	917.98 ± 11.08^a^	<0.0001
ADG, g/d per bird				
Day 0 to 7	17.08 ± 0.19^c^	17.78 ± 0.21^b^	19.09 ± 0.25^a^	<0.0001
Day 0 to 14	26.81 ± 0.65^b^	28.61 ± 0.60^b^	31.09 ± 0.53^a^	0.0015
Day 0 to 21	35.23 ± 0.78^c^	38.52 ± 0.60^b^	42.07 ± 0.62^a^	<0.0001
ADFI, g/d per bird				
Day 0 to 7	18.59 ± 0.12^a^	17.48 ± 0.37^b^	17.94 ± 0.13^ab^	0.0201
Day 0 to 14	31.21 ± 0.28^a^	29.10 ± 0.48^b^	29.90 ± 0.44^ab^	0.0206
Day 0 to 21	47.03 ± 0.67^a^	45.11 ± 0.48^b^	45.90 ± 0.53^ab^	0.0894
FI, g per pen				
Day 0 to 7	1,561.33 ± 10.42^a^	1,468.33 ± 37.38^b^	1,485.50 ± 22.80^b^	0.0285
Day 7 to 14	3,613.67 ± 60.81	3,420.00 ± 61.20	3,438.67 ± 94.47	0.1580
Day 14 to 21	6,386.33 ± 167.17	6,480.00 ± 64.74	6,528.00 ± 63.46	0.6579
Day 0 to 21	11,561.33 ± 217.87	11,368.33 ± 120.38	11,452.17 ± 158.51	0.7291
FCR				
Day 0 to 7	1.09 ± 0.01^a^	0.98 ± 0.02^b^	0.94 ± 0.01^c^	<0.0001
Day 7 to 14	1.20 ± 0.02^a^	1.03 ± 0.02^b^	0.97 ± 0.004^c^	<0.0001
Day 14 to 21	1.50 ± 0.02^a^	1.32 ± 0.007^b^	1.24 ± 0.04^b^	<0.0001
Day 0 to 21	1.32 ± 0.01^a^	1.17 ± 0.01^b^	1.10 ± 0.02^c^	<0.0001

^1^LE, low-energy diet (2,900 kcal/kg); NE, normal-energy diet (3,200 kcal/kg); HE, high-energy diet (3,500 kcal/kg).

^2^ABW, average body weight; ADG, average daily gain; ADFI, average daily feed intake; FI, feed intake; FCR, feed conversion rate.

^a,b^Mean values in a row sharing no common superscript are significantly different (*P* < 0.05).

### Effects of Dietary Energy Levels on the Plasma Parameters and Organ Index of Broilers

Compared with the control group, the chickens fed the LE and HE diets demonstrated improved urea content, in which the LE diet group had a higher content (*P* ˂ 0.05, Table 4). The TG content in the LE group was higher than that in the HE group (*P* ˂ 0.05, Table 4). Dietary energy levels demonstrated no effect on GLU, TCHO, HDL, or LDL content in the plasma of broiler chickens (*P* > 0.1, Table 4).

**Table 4.** Effects of dietary energy levels on the plasma parameters of broiler chickens^1^

	LE	NE	HE	*P*-value
UREA, mmol/L	0.75 ± 0.04^a^	0.50 ± 0.02^c^	0.61 ± 0.02^b^	<0.0001
GLU, mmol/L	14.08 ± 0.20	13.83 ± 0.26	13.67 ± 0.35	0.5809
TG, mmol/L	0.33 ± 0.05^a^	0.24 ± 0.03^ab^	0.21 ± 0.02^b^	0.0678
TCHO, mmol/L	2.87 ± 0.11	2.80 ± 0.14	2.91 ± 0.17	0.8527
HDL, mmol/L	1.75 ± 0.08	1.70 ± 0.09	1.81 ± 0.09	0.6614
LDL, mmol/L	0.30 ± 0.03	0.26 ± 0.04	0.28 ± 0.03	0.6880

^1^LE, low-energy diet (2,900 kcal/kg); NE, normal-energy diet (3,200 kcal/kg); HE, high-energy diet (3,500 kcal/kg); GLU, glucose; TG, triglycerides; TCHO, total cholesterol; HDL, high-density lipoprotein cholesterol; LDL, low-density lipoprotein cholesterol.

^a,b^Mean values in a row sharing no common superscript are significantly different (*P* < 0.05).

Compared with the control group, HE and LE treatment significantly reduced the spleen index of 21-d-old broiler chickens (*P* < 0.05, Table 5). The abdominal fat rate increased with the increase in dietary energy level (0.05 < *P* < 0.1, Table 5). Dietary energy treatment demonstrated no significant effect on the heart, liver, gallbladder, or bursa indexes (*P* > 0.1, Table 5).

**Table 5.** Effects of dietary energy levels on the organ indexes of broiler chickens^1^

	LE	NE	HE	*P*-value
Abdominal fat	1.05 ± 0.09	1.28 ± 0.08	1.33 ± 0.10	0.0927
Heart	0.44 ± 0.01	0.48 ± 0.02	0.49 ± 0.03	0.1231
Liver	2.80 ± 0.10	3.08 ± 0.16	2.99 ± 0.12	0.2861
Spleen	0.07 ± 0.004^b^	0.10 ± 0.016^a^	0.06 ± 0.006^b^	0.0211
Gallbladder	0.048 ± 0.006	0.065 ± 0.013	0.06 ± 0.009	0.4231
Bursa of Fabricius	0.20 ± 0.02	0.22 ± 0.03	0.24 ± 0.02	0.3975

^1^LE, low-energy diet (2,900 kcal/kg); NE, normal-energy diet (3,200 kcal/kg); HE, high-energy diet (3,500 kcal/kg); The organ index was a percentage to BW and expressed as %.

^a,b^Mean values in a row sharing no common superscript are significantly different (*P* < 0.05).

### Effects of Dietary Energy Level on Appetite and Central AMPK in Broiler Chickens

**Figure 1.**
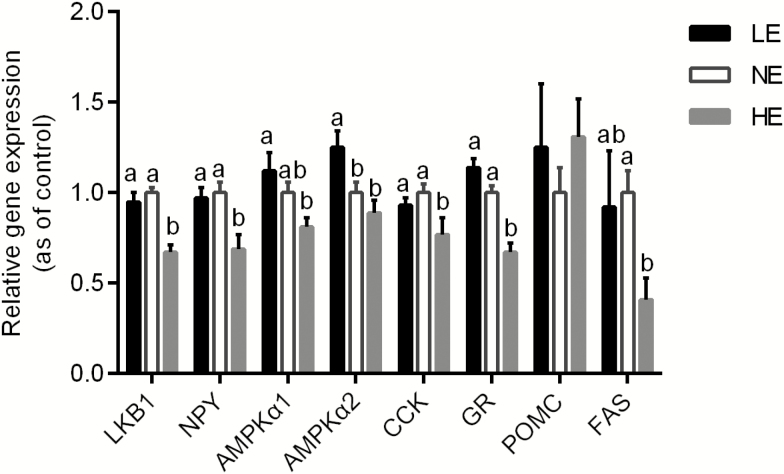
Effects of dietary energy level on the mRNA expression of *LKB1*, *NPY*, *AMPKα1*, *AMPKα2*, *CCK*, *GR*, *POMC*, and *FAS* in the hypothalami of 21-d-old broiler chickens. Values were obtained from duplicates of each sample; values are means ± SEM (*n* = 8); ^a,b^means with different letters differ significantly (*P* < 0.05) as shown by ANOVA. *LKB1*, liver kinase B1; *GR*, glucocorticoid receptor; *AMPKα1*, AMPK alpha 1 subunit; *AMPKα2*, AMPK alpha 2 subunit; *NPY*, neuropeptide Y; *POMC*, proopiomelanocortin; *CCK*, cholecystokinin; *FAS*, fatty acid synthase.

The gene expression levels of liver kinase B1 (**LKB1**), neuropeptide Y (**NPY**), cholecystokinin (**CCK**), fatty acid synthase (**FAS**), and glucocorticoid receptor (**GR**) in the hypothalamus of the 21-d-old broilers in the HE group were significantly downregulated compared with those in the control group (*P* < 0.05, Fig. 1). The HE treatment had no significant effect on the mRNA level of AMP-activated protein kinase α1 (**AMPKα1**) or AMP-activated protein kinase α2 (**AMPKα2**) compared with the control (*P* > 0.1, Fig. 1). Low-energy treatment significantly increased the gene expression of AMPKα2 in the hypothalamus of 21-d-old broiler chickens (*P* < 0.05, Fig. 1) and demonstrated no significant effect on the mRNA levels of other genes (*P* > 0.1, Fig. 1). Compared to the HE group, the LE group showed obviously upregulated AMPKα1 expression in the hypothalamus (*P* < 0.05, Fig. 1). Dietary energy level exerted no effect on the gene expression of proopiomelanocortin (**POMC**) in broilers (*P* > 0.1, Fig. 1).

## Discussion

### Effects of Dietary Energy Level on the Performance, Plasma Parameters, and Organ Index of Broiler Chickens

Appetite is largely dependent on the interoceptive sense of whether energy levels are adequate. When energy levels are perceived as adequate, feeding drive is low, and when energy levels are perceived as inadequate, the feeding drive kicks into high gear (Lora et al., 2017). The homeostatic control of appetite is mediated by the biological need to maintain the body’s energy stores (Joanne et al., 2012). In our study, the ADFI of the LE group was higher than that of the control group. This result is consistent with research on poultry (Liu et al., 2017) and mammals (Murtaugh et al., 2007). Feed intake increased with decreased nutrient density, supporting the prevailing viewpoint that FI in broilers is regulated on the basis of nutrient density (Nielsen, 2004). The difference between groups is probably due to the dietary energy deficiency that promotes feeding behavior to meet the body’s energy needs. These results show that broilers can adjust the feed intake according to the energy state of the diet and are consistent with previous research (Yuan et al., 2008; Liu et al., 2017). Feeding on the HE diets increased body weight gain, which is in agreement with studies in humans and animals (West and York, 1998; Murtaugh et al., 2007; Yuan et al., 2008). In addition, early studies on broilers have found that HE diets improved broiler BW gain (Grover et al., 1972; Reece and McNaughton, 1982; Zhao et al., 2009). The favorable effect of HE on BW gain should be ascribed to the high energy ingestion. On the other hand, a high energy level in the diet may also increase lipid accumulation in the bodies of broilers (Deaton and Lott, 1985; Summers et al., 1992; Nahashon et al., 2005). Yuan et al. (2008) found that HED chickens had significantly higher plasma insulin levels and that dietary energy levels were associated with impaired glucose-insulin balance. Unfortunately, in this study, we did not measure the insulin level in plasma, but we suspected that the body weight gain caused by a HE diet may be related to insulin imbalance.

In this study, we found that the abdominal fat rate (as a percentage of BW) presented a gradually increasing trend with increasing dietary energy level, indicating that the HE level in the diet promoted abdominal fat deposition. Some studies have found that the contents of BW gain changed toward a higher proportion of adipose tissue rather than skeletal muscle in HED treatment (Yuan et al., 2008). Overall, high energy intake is the most fundamental reason (Eits et al., 2002). In addition, compared with carbohydrate and protein in diet, dietary fat is easier to deposit (Lean and James, 1988; Horton et al., 1995). Surprisingly, in this study, we found that LE and HE treatments significantly reduced the spleen index, which might indicate that the spleen is more sensitive to metabolic disorders.

Blood biochemical parameters can reflect the function and metabolism of nutrients in the body (He et al., 2015; Liu et al., 2015). Blood urea nitrogen (**BUN**) is the main end product of protein metabolism in vivo, and decreased BUN indicates that more proteins are being synthesized (Kim et al., 2013). The results of this study showed that the plasma urea was significantly increased on the HE and LE diets, indicating that a large proportion of proteins in chickens are catabolic. Lipid accumulation in cells depends on the balance between lipogenesis and lipolysis of TG, which is controlled by nutrition and hormones (Kersten, 2001). The concentration of TG in serum may reflect the status of lipid metabolism in the body (He et al., 2015). In this study, we found that the plasma TG was significantly increased in the LE diet compared with the HE group, reflecting that a high dietary energy level promoted fat deposition and that the LE diet lessened lipid accumulation. These results show that reserved energy is mobilized when the energy content in the diet is low and cannot meet the growth requirements of the body for the maintenance of normal physiological functions.

### Effects of Dietary Energy Level on Appetite and Central AMPK in Broiler Chickens

Two key appetite-regulating neuronal populations in ARC regulate nutritional status signals and influence energy homeostasis in birds. One population of neurons suppresses appetite through the release of POMC, as well as cocaine- and amphetamine-regulated transcript (Boswell, 2005; Cai et al., 2015), whereas the other group of neurons stimulates appetite through the release of NPY and agouti-related peptide (Broberger et al., 1998; Romana et al., 2013). In our study, the results showed that the gene expression of NPY in the hypothalamus of broilers fed the HE diet was significantly lower than that in the control group, and the mRNA level of POMC demonstrated no significant change, suggesting that the appetite of chickens fed the HE diet was markedly suppressed through the inhibition of NPY expression. However, there was no change in feed intake, probably because the increased BW gain concealed the suppressing effect of the HE diet.

The effect of glucocorticoids is mediated by ligand-dependent activation of GR, which is expressed in almost every cell type. Activated GR acts as a transcription factor that controls the level of target gene expression and modulates intracellular signaling pathways (Herrlich, 2001; Karin and Chang, 2001; Hafezi-Moghadam et al., 2002). In this study, we found that GR transcription was decreased in the HE group, and thus, the GR pathway was blocked. Many studies have shown that stress increases GR gene expression in birds, accompanied by weight loss (Hu et al., 2019). Our results showed that a HE diet could reduce the production of glucocorticoids and alleviate environmental stress in the same feeding environment. Fatty acid synthase, a main lipogenic enzyme (Li et al., 2016), can alter the rates of biosynthesis and hydrolysis of fatty acids (Smith et al., 2003). Fatty acid synthase catalyzes the last step in the fatty acid biosynthetic pathway, which is believed to be a determinant of the maximal capacity of a tissue, liver in particular, to synthesize fatty acids by de novo lipogenesis (Postic and Girard, 2008). The results of this study showed that dietary HE treatment significantly downregulated the gene expression of FAS in the hypothalamus compared with the controls. These results may be related to tissue-specific regulation of FAS gene expression; however, its mechanism needs to be further studied.

Gut hormones are believed to contribute to short-term feelings of satiety and hunger (Badman and Flier, 2005), which may reduce food intake by decreasing hypothalamic orexigenic signaling and increasing anorectic signaling (Batterham et al., 2002; Jobst et al., 2004). Cholecystokinin, a gastrointestinal hormone, is released from the proximal intestine following nutrient ingestion, particularly fat, and acts as a signal of satiation, reducing meal size by suppressing orexigenic neurons (Matzinger et al., 1999; Bi and Moran, 2002). Cholecystokinin is a gut peptide that has long been established as a postprandial satiety signal and an anorexic factor in avian species (Panchanathan et al., 2006). Liu et al. (2012) found that a decrease in feed intake in corticosterone-exposed hens was not related to the central or peripheral expression level of CCK. In this study, we found that the gene expression of hypothalamic CCK was significantly decreased by feeding on the HE diet. Obviously, this result is inconsistent with the anorexic effect of CCK. It should be noted that the differences in the translation rate of CCK mRNA are still unknown, and its activity may depend on differential posttranslational processing rather than differential expression (Sayegh et al., 2014).

Adenosine monophosphate-activated protein kinase is a ubiquitously expressed kinase found in a number of important hypothalamic nuclei, including ARC, ventral medial hypothalamus (VMH), paraventricular nucleus (PVN), and lateral hypothalamus (LH) (Minokoshi et al., 2004; López et al., 2008). Once phosphorylated on threonine 172 in the α-catalytic subunit, AMPK activity increases, and then it functions as an intracellular energy sensor, switching off ATP-consuming pathways and switching on ATP-producing pathways, such as glucose uptake and fatty acid oxidation (Steinberg and Kemp, 2009). Liver kinase B1 is a major AMPK kinase that is constitutively active and phosphorylates AMPK at the Thr172 site of the subunit (Hawley et al., 1996, 2003; Hong et al., 2003; Woods et al., 2003; Shaw et al., 2004). Kohno et al. (2011) found that AMPK activates NPY neurons in the hypothalamic ARC to increase food intake in rats, which is consistent with our findings. In our study, HE diet treatment significantly decreased LKB1 and AMPKα1 gene expression in the hypothalamus compared to the control group. At the same time, LE diet treatment significantly increased the gene expression of AMPKα2. These results revealed that the central AMPK signal pathway was restrained by the administration of the HE diet, which restrained lipid oxidation and promoted lipid synthesis. Conversely, the central AMPK signaling pathway in chickens fed the LE diet was activated, promoting catabolism and generating more energy.

In conclusion, the HE diet suppressed the appetite and the AMPK signal pathway in the hypothalamus, whereas the LE diet activated central AMPK.

## Funding

This work was supported by the National Key R&D Program of China (2018YFD0501401-3), the National Natural Science Foundation of China (31472115), the Shandong Province Agricultural Industry Technology (SDAIT-11-08), and Funds of the Shandong “Double Tops” program.

## Conflict of Interest

None Declared.
